# Portal Vein Thrombosis Secondary to COVID-19: A Rare Complication

**DOI:** 10.7759/cureus.22780

**Published:** 2022-03-02

**Authors:** Khushboo K Agarwal, Moiuz Chaudhri, Vistasp J Daruwalla, Arif Saleh, Eric Costanzo

**Affiliations:** 1 Internal Medicine, Jersey Shore University Medical Center, Neptune City, USA; 2 Internal Medicine, Riverview Medical Center, Red Bank, USA; 3 Internal Medicine, Shore Pulmonary, Ocean, USA; 4 Interventional Radiology, Radiology Associates, Laural, USA; 5 internal medicine, Jersey Shore University Medical Center, Neptune City, USA

**Keywords:** anti coagulation, acute portal vein thrombosis, portal vein thrombosis, rare association, complication, covid 19

## Abstract

The novel coronavirus disease 2019 (COVID-19) pandemic has created a lasting impact in the world. It presents with various clinical manifestations, ranging from an asymptomatic state to respiratory system abnormalities, multi-organ involvement, thrombosis, and death. This case describes a 46-year-old female presenting with intractable abdominal pain leading to portal vein thrombosis (PVT), a diagnosis attributed to an unresolved COVID-19 infection.

## Introduction

Coronavirus disease 2019 (COVID-19), which emerged in Wuhan, China, at the end of 2019, has been classified as a pandemic. The virus has impacted nearly 240 million people across the globe, ranging from an asymptomatic carrier state to respiratory symptoms, cardiovascular abnormalities, neuropsychiatric symptoms, hematologic manifestation, particularly portal vein thrombosis (PVT), and multiorgan failure to death [1-2]. The purpose of this report/study is to increase awareness of this rare complication of COVID-19 (PVT).

## Case presentation

**Figure 1:**
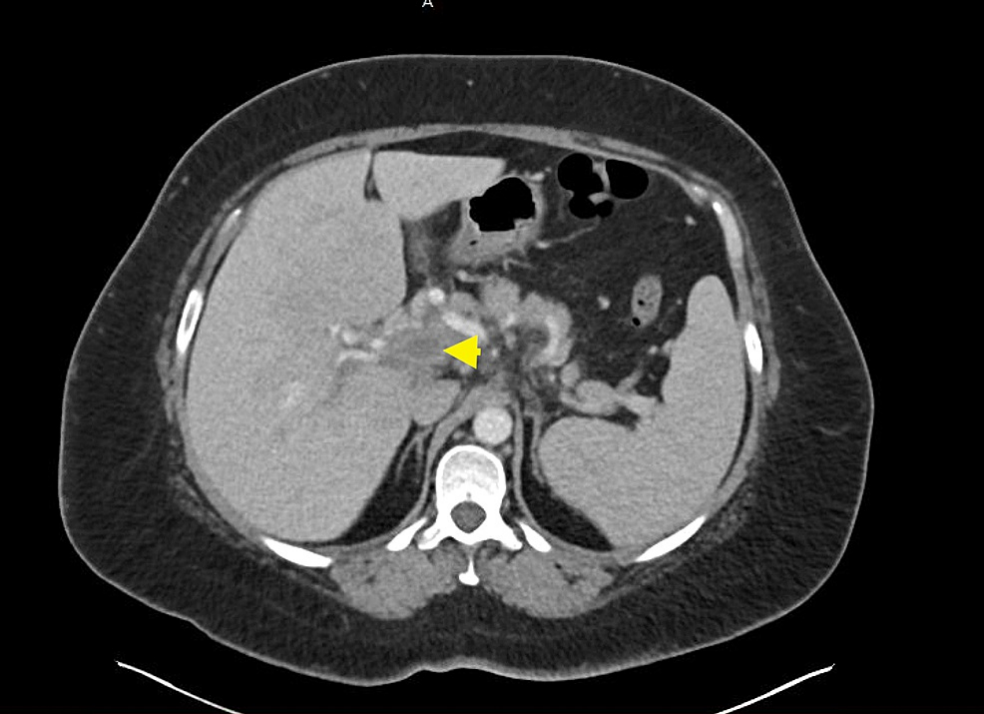
Main portal vein thrombosis (yellow arrow)

A 46-year-old female with a past medical history of Wegener's granulomatosis status post-Rituxan, thromboembolism of port site status post-rivaroxaban for six months, hypertension, and recently diagnosed resolving COVID-19 pneumonia presented to the emergency department (ED) for worsening abdominal pain. In the ED, the patient underwent a computed tomography (CT) scan of the abdomen and pelvis with contrast depicting portal vein thrombosis and superior mesenteric vein thrombosis with thrombophlebitis (Figure 1). The physical examination was unremarkable except for diffuse abdominal pain without distention. The patient was started on intravenous anticoagulation for a week and was transitioned to oral anticoagulation.

## Discussion

The novel coronavirus disease 2019 pandemic has impacted nearly 240 million people from at least 223 countries throughout the globe. This pandemic has led to more than 4.6 million fatalities worldwide, with the death toll increasing daily [3]. The clinical manifestation of COVID-19 varies among the patients due to the immune system, gender, age, and other factors. General symptoms like cough and fever are common, but numerous complications like respiratory, cardiovascular, and musculoskeletal issues and thrombosis are less common [4]. A meta-analysis of 50,466 patients with COVID-19 found that 96.6% of the patients had abnormal CT scans, a finding found in our patient [2,5]. The literature suggests that COVID-19 increases the risk of coagulation abnormalities, particularly venous thromboembolism (VTE), disseminated intravascular coagulation (DIC), deep vein thrombosis (DVT), and portal vein thrombosis (PVT) [2,6].

A literature review and clinical evidence suggest that PVT is a rare abnormal complication of COVID-19 presenting with abdominal pain. According to the literature, the coagulation pathway may be activated by COVID-19 infection due to the inflammatory response of cytokines to the virus [6-7]. Cytokines such as interleukin-6 (IL-6) can increase the expression of tissue factor (TF), thus facilitating the process of clot formation. Clinical evidence suggests that Wegener’s granulomatosis is associated with an increased risk of thrombosis, evident by Wegener’s granulomatosis etanercept trial (WGET). The trial reported an increased incidence of VTE in 180 participants. A different study from the Netherlands evaluated thrombosis in a cohort of 198 patients and found an incidence rate of 1.8 events per 100 person-years [8]. Since COVID-19 is a well-documented state of hypercoagulability, along with our patient's history of thromboembolism, portal vein thrombosis can be attributed to the unresolved COVID-19 infection in our patient.

## Conclusions

A COVID-19 infection can present with many variations that can be challenging to diagnose. In a patient with severe abdominal pain and unresolving COVID-19 disease, it is crucial to consider portal vein thrombosis as a potential diagnosis.
